# Acceptability of Telemedicine Features to Promote Its Uptake in Practice: A Survey of Community Telemental Health Providers

**DOI:** 10.3390/ijerph17228525

**Published:** 2020-11-17

**Authors:** Brian E. Bunnell, Janelle F. Barrera, Samantha R. Paige, Dylan Turner, Brandon M. Welch

**Affiliations:** 1 Department of Psychiatry and Behavioral Neurosciences, Morsani College of Medicine, University of South Florida, Tampa, FL 33612, USA; jfb@usf.edu; 2 Doxy.me, LLC, Rochester, NY 14623, USA; samantha.paige@doxy.me (S.R.P.); dylan@doxy.me (D.T.); brandon@doxy.me (B.M.W.); 3 Biomedical Informatics Center, College of Medicine, Medical University of South Carolina, Charleston, SC 29403, USA

**Keywords:** telehealth, telemedicine, mobile health, mental health, technology acceptance

## Abstract

Understanding what motivates mental health providers to use telemedicine (i.e., telemental health) is critical for optimizing its uptake, especially during unprecedented times (e.g., the COVID-19 pandemic). Drawing from the Technology Acceptance Model (TAM), this report examined the characteristics of telemental health providers and how the acceptability of telemedicine features contributes to their intention to use the technology more often in practice. Telemental health providers (*N* = 177) completed an online survey between March and May 2019. Most providers (75%) spent less than 25% of their work-week using telemedicine, but 70% reported an intention to use telemedicine more in the future. The belief that telemedicine affords greater access to patients, work-life balance, flexibility in providing care, and the opportunity to be at the forefront of innovative care were significant predictors of intentions to use the technology more in the future. Other significant predictors included needing assistance to coordinate insurance reimbursements, manage a successful telemedicine practice, and integrate the telemedicine program with other health IT software. Findings have important implications for increasing the frequency of telemedicine use among telemental health providers. Future research and practice should leverage providers’ positive beliefs about telemedicine acceptability and consider their needs to enhance its uptake.

## 1. Introduction

Widespread Internet access and advancements in technology have revolutionized how healthcare is delivered to prevent and manage the leading causes of death and disability worldwide. Mobile health (mHealth) technologies, for example, support non-clinical care delivery (e.g., health education and data reporting and monitoring) as well as clinical intervention between patients and providers [1]. This clinical application, termed telemedicine, leverages information communication technology to enable providers to remotely deliver healthcare services and consultations to patients through video conferencing, among other modalities [2]. Alongside growing evidence for its clinical and cost-effectiveness, telemedicine has changed the way that healthcare is practiced in unprecedented times, such as the COVID-19 pandemic [3,4].

Telemedicine supports the provision of healthcare for a variety of conditions (e.g., diabetes, hypertension, heart failure, cancer) [5,6,7,8,9], but its potential to shape the delivery of mental health care is undeniable. Systematic and meta-analytic reviews have demonstrated that mental health treatment delivered via telemedicine, or telemental health, is equally, and in some cases more, effective than treatment delivered in-person and results in higher patient satisfaction at reduced costs [10,11,12,13]. The effectiveness and satisfaction of telemental health treatment is likely to be due to its ability to alleviate physical (e.g., travel time, missed work, and financial costs) and socio-cultural (e.g., concerns relating to stigma) barriers that are notorious for impeding healthcare accessibility and continuity [14,15,16,17,18].

In a review of Medicare fee-for-service claims between 2014 and 2016, psychiatrist telemental health providers were more likely to be early in their careers, employed in large practices, and serving rural regions as compared with psychiatrists who did not use telemental health services [19]. Despite this knowledge, little is known about the characteristics of community telemental health providers, including their use of telemedicine and the factors that motivate them to use the technology [20,21,22]. To date, only two studies have surveyed mental health providers about their use and perceptions of telemedicine. Simms et al. [23] surveyed Canadian mental health providers, half of whom had been practicing for 10–20+ years. This survey found that only 40% had provided telemental health (i.e., video conferencing) to their patients and that these services were provided about once per year, on average. Of those providers, 75% did not receive training in how to provide telemental health services. Having been practicing for longer, previously receiving telemental health training, and perceiving the technology to be easy-to-use were also associated with more frequent telemedicine use. In another study, Gershkovich et al. [24] surveyed a sample of mental health providers recruited through listservs and mailing lists of state, national, and international behavioral health organizations. Approximately 72% of the providers used remote patient services (i.e., telephone, email, videoconferencing, instant messaging, and free-standing websites). However, only ~27% had used videoconferencing (i.e., 16% of the entire sample), with less than 10% reporting regular use. Despite this promising evidence, the diffusion and uptake of telemental health care in community mental health clinics has lagged [25,26,27]. There is a need to understand the trends in telemental health use, as well as the characteristics of mental health providers who embrace its use in practice. This includes providers other than psychiatrists, including mental health counselors and social workers. 

Mental health providers are “gatekeepers’’ to the implementation of telemedicine in every-day clinical practice [27,28,29]. A commonly cited factor that hinders the uptake of telemental health visits includes slow-shifting public policies that leave concerns about being reimbursed by health insurance companies [25]. This is likely to be why less than 20% of telemental health providers eventually bill insurance companies for providing the service [24]. Given the potential of telemedicine to alleviate disparities in accessing community mental health treatment, empirical evidence is needed to describe factors that contribute to telemental health providers’ acceptability and the ultimate uptake of their telemedicine platform in clinical practice. 

Drawing from the tenets of the Technology Acceptance Model (TAM), an individual’s intention to use a technology is driven by its perceived acceptability, which is broadly conceptualized according to the user’s beliefs about its usefulness and ease-of-use [30]. A recent systematic review found that the TAM is the most widely used model to guide technology acceptance research in telemedicine, which is due to its simplicity and capacity to explain the high amount of variance in data (*Mdn R^2^ =* 0.68) [31]. Results of the review further demonstrated that the perceived usefulness of telemedicine has the strongest predictive power in telemedicine uptake intentions among healthcare providers. For healthcare providers, the perceived usefulness of telemedicine is dependent on intentions about its efficiency to streamline diagnosis, disease monitoring, and treatment [31]. 

In prior research, about 50% of mental health providers rate telemental health visits as very/somewhat useful and 34% rated it to be somewhat easy/very easy to use [23]. According to Gershkovich et al. [24], commonly endorsed beliefs that reinforce the use of telemental health services include the ability to reach clients from a distance, convenience for clients and therapists, real-time monitoring and intervention, increased access to specialized care, and low-cost services. On the other hand, common challenges that disengage positive beliefs about telemedicine include limited personal connections due to mediated contexts, addressing technological difficulties, uncertainty about legal issues, privacy concerns, and challenges responding to crisis situations. As such, the acceptability of telemental health includes positive and negative beliefs about its features. There is a significant need to understand user acceptance according to the features that motivate its initial use and features that need improvement to secure long-term use by providers. This evidence will inform more precise intervention to support provider acceptance, uptake, and sustained use of telemedicine over time.

The purpose of this report is to describe the characteristics of community telemental health providers, with strong attention paid to better understanding which telemedicine features characterize its acceptance and frequent use in the future. A secondary aim is to provide a pre-COVID-19 pandemic baseline assessment for various aspects of the acceptability of these features. Telemedicine was quickly identified as a healthcare delivery solution, while risk mitigation guidelines were implemented [3,32]. With statements suggesting that telemedicine will remain an integral aspect of healthcare delivery post-COVID-19 [33], it becomes valuable to document baseline data for monitoring trends regarding the beliefs about telemedicine features in this profession. Findings of this report have important practical and theoretical implications for increasing the frequency of telemedicine uptake in clinical practice. 

## 2. Materials and Methods 

### 2.1. Sample and Procedures

This is a retrospective review of cross-sectional, de-identified data from a 15-min web-based marketing survey administered among practicing community telemental health providers registered with doxy.me (https://doxy.me/), a HIPAA-compliant telemedicine solution with more than 700,000 active provider users. Between 25 March 2019 and 1 May 2019, providers who indicated mental/behavioral health as their clinical specialty under their doxy.me profile received an email invitation to complete the survey. Of the 1944 providers who were sent email invitations, 290 (14.9%) started the survey and 177 (9.1%) completed it. Prior to the survey, providers were directed to an electronic consent form where they indicated their agreement to have their de-identified data used for marketing research and publication. Compensation was not offered, and the Institutional Review Board at the University of South Florida determined that use of this data for publication does not constitute research involving human subjects.

### 2.2. Measures

The survey was developed through an iterative process with marketing and leadership teams based on their knowledge and experience of telemedicine, consultation with a licensed clinical psychologist with telemedicine experience (i.e., the first author), and prior relevant survey studies [34,35]. It was also reviewed for readability, relevance to the mission of the company, and efficiency of completion. Survey items included demographic (e.g., gender, race, ethnicity) and professional questions (e.g., highest level of education achieved, mental health specialty and setting, the proportion of time spent providing telemental health services, health insurance billing practices, reason for integrating telemedicine into their current practice). Next, providers were asked to indicate which features of telemedicine serve as benefits (e.g., can see patients wherever they are, can have a better work-life balance) and barriers (e.g., security/privacy concerns, reimbursement issues) to their practice. The selection of each benefit and barrier was measured dichotomously, where 1 = yes and 0 = no. Next, providers were asked to select the reasons for using their current telemedicine platform, the telemedicine features that are most important to their practice, and the telemedicine features that need to be improved. For each of the aforementioned variables, providers were presented with a predetermined list of telemedicine features (e.g., HIPAA compliance, low cost, no downloads) and asked to select all that apply, where 1 = yes and 0 = no. Finally, providers were asked about their intention to use telemedicine in the future with three response options (i.e., I expect to use it “less often, the same, or more”). Only *n* = 1 selected “I expect to use it less often.” As a dependent variable for our logistic regression model, this participant was excluded and the variable was reduced to “I expect to use it about the same” = 0 and “I expect to use it more” = 1. 

### 2.3. Data Analysis

We used SPSS Version 27.0 (Armonk, NY: IBM Corp.) to describe the demographic and professional characteristics of the sample. To address the aims of the report, we also computed descriptive statistics to examine variables intended to describe telemedicine acceptability (i.e., features that are benefits or barriers of telemedicine, features contributing to the decision to use telemedicine, telemedicine features that are important, and telemedicine features that could be improved) and intentions for using telemedicine more often in the future. We conducted five multiple logistic regression analyses to examine the association between intentions to use telemedicine in the future (0 = the same amount; 1 = more often) and: (1) benefits of using telemedicine; (2) barriers of using telemedicine; (3) reasons for using current telemedicine platform; (4) important telemedicine features; and (5) telemedicine features that need improvement. Each regression analysis was a single-step model. Tolerance and variation inflation factors (VIF) were consisted to ensure that multi-collinearity was not a threat to model interpretation. There was a statistically non-significant (*p* > 0.05) association between demographic factors and intentions to use telemedicine more often in the future; therefore, the models did not include these variables as covariates. 

## 3. Results

### 3.1. Telemental Health Provider Characteristics

Table 1 shows provider demographics. Community telemental health providers were, on average, 46.4 years old (*SD* = 12.2 years; min = 26 and max = 83). The majority of providers identified as female (68.9%), white (78%) and non-Hispanic (90.4%). Providers had been providing mental health care for an average of 13.6 years (*SD* = 10.4; min = 1 and max = 49 years) and had been telemental health providers for an average of 2.1 years (*SD* = 1.9; min = < 1 and max = 10). Roughly two-thirds had obtained a master’s degree (63.8%) and 25.4% had received a doctoral degree (i.e., PhD or PsyD). 

Most of the telemental health providers were employed by individual practices (69.5%) or small clinics (18.1%). Among providers who were employed by individual practices, most were mental health counselors (*n* = 37; 30.1%), psychologists (*n* = 32; 26%) or social workers (*n* = 27; 22%), whereas less than 4% were primary care providers, psychiatrists, or health coaches. A similar trend existed for small clinics, with the majority of providers being mental health counselors (*n* = 14; 43.8%), psychologists (*n* = 5; 15.6%), or social workers (*n* = 6; 18.8%).

### 3.2. Characteristics of Telemedicine Use in Clinical Practice

Table 2 demonstrates that nearly 85% of providers spent over a quarter of their work-week providing mental health care to patients; only about 25% spent over a quarter of this time providing telemental health care. A combination of in-person and telemedicine sessions (44.1%) or only using telemedicine when necessary (46.9%) were reported by providers. Only half (50.6%) of the providers reported billing health insurance companies for telemental health visits. Not shown in Table 2, providers who billed insurance companies were mental health counselors (38.9%), social workers (24.4%), and clinical psychologists in independent practices (69.5%) and clinics (18.1%). 

### 3.3. Perspectives on Telemedicine Use in Mental Health Care: Perceived Benefits and Barriers

Table 3 shows that over half of the community telemental health providers identified seeing patients wherever they are (72.3%) and reaching patients who otherwise wouldn’t receive care (67.2%) as benefits of telemedicine. Over half of the providers reported barriers related to technological problems (65.5%), whereas fewer (29.4%) identified patient satisfaction barriers

### 3.4. Reasons for Using Telemedicine, Important Features, and Intentions for its Future Use

Table 4 shows that over half of the providers chose to use telemedicine because it is HIPAA compliant (88.7%) and cheaper than other solutions (56.5%). The most important features included HIPAA compliance/security (81.4%), free/low cost (67.2%), and being easy for patients to use (62.7%). Less than 50% of the providers identified a feature that should be improved to enhance the telemedicine experience. Audio/video quality and technical issues were the most commonly reported features needing improvement (identified by 48% and 37.3% of providers, respectively). Finally, over 70% of the telemental health providers stated that they intend to use telemedicine more in the future. 

### 3.5. Association of Telemedicine Acceptance Features and Intentions for More Frequent Use in the Future

Table 5 includes the results of a statistically significant multiple logistic regression model, examining the relationship between benefits of telemedicine and intentions to use telemedicine more in the future (χ^2^ = 14.98, *df* = 7, *p* < 0.05; Nagelkerke *R*^2^ = 0.12). Providers who identified the following benefits of telemedicine had higher odds of intending to use it more in the future, compared with providers who did not indicate the same benefits. These benefits include: reaching patients who otherwise wouldn’t receive care (odds ratio (OR) = 5.99, *p* < 0.05); having a better work-life balance (OR = 7.13, *p* < 0.05); being at the forefront of innovative care (OR = 5.83, *p* < 0.05); seeing more patients and expand my practice (OR = 9.27, *p* < 0.001); providing the care the way I want (OR = 6.46, *p* < 0.05); and cutting my expenses (OR = 7.40, *p* < 0.05).

A subsequent analysis was conducted to examine the relationship between barriers to using telemedicine in practice and intentions to use it more in the future; however, the model resulted in poor fit (χ^2^ = 7.68, *df* = 5, *p* = 0.18; Nagelkerke *R*^2^ = 0.06). We also examined the reasons why telemental health providers decided to use their current telemedicine platform and the relationship with intentions to use it more in the future, and the model approached good fit (χ^2^ = 15.14, *df* = 8, *p* = 0.06; Nagelkerke *R*^2^ = 0.12). Providers who intended to use telemedicine more in the future chose their current platform because it was easier than other solutions (OR = 3.20; 95% CI = 1.02–9.98; *p* = 0.05), used and recommended by colleagues (OR = 2.59; 95% CI = 0.91–7.34; *p* = 0.07), and had great customer support (OR = 5.18; 95% CI = 0.86–31.14; *p* = 0.07).

Table 6 includes the results of a statistically significant multiple logistic regression model, examining the relationship between telemedicine features that could be improved and intentions to use telemedicine more in the future (χ^2^ = 38.55, *df* = 11, *p* < 0.001; Nagelkerke *R*^2^ = 0.28). Providers who recommended the following improvements had higher odds of intending to use telemedicine more in the future, compared to providers who did not recommend the same improvements. These include: helping coordinate health insurance reimbursements (OR = 4.29, *p* < 0.05); assisting in becoming more successful in their telemedicine practice (OR = 13.17, *p* < 0.05); and integrating the platform with other health IT software (OR = 5.45, *p* < 0.05). We also examined the relationship between important telemedicine features and intentions to use telemedicine more in the future, but the model had poor fit (χ^2^ = 5.81, *df* = 7, *p* = 0.56; Nagelkerke *R*^2^ = 0.05) and was not pursued further.

## 4. Discussion

The purpose of this report was to describe the characteristics of telemental health providers and telemedicine features that contribute to their intentions to use it more often in clinical practice. Despite being registered users of a telemedicine platform, the providers in this report did not frequently use the platform for telemental health visits. Nearly 75% of the providers used telemedicine to deliver telemental health care <25% of the time they interacted with patients. Providers held positive beliefs about the capacity of telemedicine to increase patient access to care, yet they also reported perceived barriers about patient security and technological challenges. Most providers reported that HIPAA compliance was a strong motivator and important feature of their current telemedicine platform. However, positive beliefs about telemedicine and the need for more specialized support were strong predictors of intentions to more frequently use telemedicine in practice. In addition to providing a baseline assessment for the acceptability of telemedicine pre-COVID-19, this report provides formative data to inform interventions to increase the uptake and frequent use of telemental health services. 

### 4.1. Principal Findings

The majority of community telemental health providers surveyed in this report were female, white, non-Hispanic, licensed mental health counselors and social workers employed within individual practice settings or small clinics. These characteristics are similar to those observed in the overall workforce in the US [36,37]. Half of the providers reported that they billed health insurance companies for telemental health visits, the majority of whom were master’s-level providers in individual practice. This was much higher than in the study by Gershkovich et al. [24], who found that 15.8% of providers reported billing insurance for telemental health visits. One reason for increases in insurance billing may be a result of updates in telehealth regulations and policies [38]. Even with the recent policy improvements, however, barriers still exist that prevent patients who do not live in rural areas or are not covered by Medicare to access affordable telehealth services [38,39]. Evidence to demonstrate the clinical and patient-reported benefits of telemental health care among rural and non-rural patients will be important for addressing this regulatory limitation.

A commonly reported benefit of telemedicine included increasing patient access to care and its most important feature was HIPAA compliance, followed by being low-cost and easy-to-use. Although there are regulatory standards to ensure HIPAA-compliance in data sharing and communication via telemedicine [40], about 70% of providers expressed concerns about the impact of technological problems and 40% indicated security/privacy concerns related to telemedicine. Li [41] found that the degree to which a person feels confident in their ability to manage their online personal security and privacy will influence their skills to effectively use technology to achieve their health-related goals. Therefore, greater attention must be paid to enhancing community telemental health providers’ skills to effectively use telemedicine platforms. Enhancing confidence that patients can use the technology to effectively engage with them during telemental health visits will also be important. To achieve this goal, providers must maintain transparency and fidelity when interacting with patients during these visits [42]. Telemedicine platforms may also serve a role in providing reassurance to both patients and providers by indicating whether or not its features are evidence-based and had undergone user-centered design testing.

Consistent with technology adoption models [30], telemental health providers selected their current telemedicine platform if it included features that were important to their practice. Interestingly, these features did not reinforce providers’ intentions to use telemental health services more often in the future. Instead, the perceived benefits of using telemedicine and the desired need to improve the workflow of telemental health services were associated with intentions for its more frequent use in the future. These workflow improvements included needing assistance to coordinate health insurance reimbursements, facilitate a successful telemedicine practice, and integrate the telemedicine program with existing health IT solutions. This finding reflects a socio-ecological perspective towards telemedicine adoption [43], supporting that its acceptance includes providers’ beliefs about its usefulness and ease-of-use, as well as the capacity of their healthcare organization to facilitate its successful implementation in practice. 

### 4.2. Limitations

This report includes a secondary analysis of de-identified marketing data from a commercial telemedicine company, meaning we had little control over the sampling and recruitment processes. First, data were collected using a purposive sampling approach. Because there was no sampling frame, potentially knowledgeable and appropriate individuals may have been excluded. We were unable to assess any bias between those who responded to the survey and those who did not respond, because we did not draw from an already constructed sampling frame. Although this raises concerns about the external validity of the results, we note above that the socio-demographic characteristics of our sample is representative of the workforce in the US. Findings should be explored and confirmed among telemental health providers who broadly use telehealth programs, including other telemedicine platforms. A second limitation that contributes to the generalizability of results is the low survey response rate. Although this rate is consistent with the average response rates of other web-based surveys [44], our absent sampling frame and approach to recruitment may begin to explain the low response rate. A randomized sample of telemental health providers would be ideal for future research on this topic, specifically one that applies stratification procedures to ensure that various types of providers (i.e., social workers, psychologists) and their place of practice (i.e., small clinics, individual clinics) are represented. In regard to recruitment, more efforts could have been made to promote responses such as reminder emails, phone calls and texts. The confidence intervals in this study’s multiple logistic regression analyses lacked precision. However, VIFs for each independent variable across all regression analyses were less than 5.0, which is less than the recommended cut-off of 10.0 to signal serious multicollinearity between variables within a given model [45]. To better understand the relationship between telemedicine features and intentions to use the technology more in the future, future research with a larger sample of providers who affirm the desire or need for particular telemedicine features (e.g., help becoming more successful with their telemedicine practice) is needed.

### 4.3. Practical and Theoretical Implications

Although all providers who completed the survey were registered telemedicine users, the frequency with which they used the technology in practice was considerably low. Providers who indicated an interest in using telemedicine more often in the future were drawn to the patient-centric benefits of telemedicine, and desired more support in making telemedicine a central aspect of their clinical practice. The development and adaptation of telemedicine features to better support telemental health providers’ workflow may take a significant amount of time and resources. To begin making a low-cost impact on increasing how frequently mental health providers use telemedicine services, healthcare companies and organizations may consider testing message-based interventions that reinforce the perceived benefits and efficacy of using telemedicine. For example, messages could reinforce that telemental health visits increase patient access to high-quality care and highlight the value of a HIPAA-compliant telemedicine platform, if applicable. Drawing from technology adoption models and behavior change theory, such as the Transtheoretical Model of Behavior Change [46], may be especially useful in tailoring messages to those in the contemplation and preparation stages of increasing the frequency in which they use telemedicine in clinical practice. 

This study also has important theoretical implications that should be considered for future research and practice. The TAM may be a worthwhile framework for understanding the initial uptake of telemedicine [31], but the features of other technology adoption models are useful for understanding how to increase its use over time. According to a systematic review by Harst et al. [31], the Unified Theory of Acceptance and Use of Technology (UTAUT) includes facilitating conditions as a central feature of telemedicine use. Facilitating conditions are infrastructural features that support the utilization of telemedicine in healthcare systems and organizations [31]. In a recent study conducted in tele-dermatology [47], researchers found that healthcare providers’ perceptions of the infrastructural capacity of their healthcare systems (i.e., facilitators) was the strongest predictor for their intention to use the technology in the future, more so than the perceived usefulness of telemedicine and its ease-of-use. This is consistent with the results of the current study, which found that the perceived importance of various telemedicine features did not influence intentions to use the technology. Rather, telemental health providers’ desired needs for support, related to health insurance reimbursement, successful telemedicine practice management and health IT integration, predicted this future use. Results support the recommended modifications of technology acceptance models to consider system-level facilitators of technology use [43,47]. Results also provide insight about which system factors are most impactful on telemental health providers’ intentions to use telemedicine in the future.

Intervening on system-level factors related to telemedicine acceptance requires a strategic approach. One approach includes the integration of telemedicine with health IT programs, including electronic health records (EHRs), which have become integral to coordinating care across healthcare delivery systems. EHR systems increase the transparency and efficiency of healthcare coordination by streamlining how patient and insurance data are accessed, entered, and monitored by providers, among other impactful capabilities [48,49]. Healthcare providers can become advocates in this area by understanding what type of integration is best and feasible for their practice. As such, further research is needed to understand disparities in its integration across geographic regions where healthcare resources are sparse, as well as the personal and professional demographics (i.e., medical specialties and sub-specialties) of telemental health providers. 

Due to the recent acceleration of telemedicine due to COVID-19 pandemic, regulatory and financial barriers to telemedicine reimbursement have now been alleviated. For example, on 13 March 2020, the Trump Administration announced an emergency declaration under the Stafford Act and the National Emergencies Act, which led to the Center for Medicare & Medicaid Services (CMS) expanding telehealth benefits [50]. Many private insurance companies have also expanded their policy coverage on telehealth [51]. In addition, the Office for Civil Rights (OCR) enforced discretion for telehealth remote services during the COVID-19 public health emergency, resulting in ease of HIPAA restrictions with hope of good faith provision [52]. While the OCR’s enforcement discretion does relax restrictions on telehealth and may increase concern on level of security, it is only temporary for the duration of the public health emergency. This report provides a baseline assessment for telemental health care providers’ characteristics and acceptability of telemedicine in practice. Future research is needed to examine how the characteristics and use of telemedicine has changed in response to the COVID-19 pandemic, and whether or not any changes are sustained over a significant period of time.

## 5. Conclusions

This study explores the characteristics of community telemental health providers who use telemedicine, and how telemedicine features are associated with intentions to use the technology more often in the future. Although providers believed that telemental health increases patient access to care, they also had concerns about patient security, safety, and technology challenges. Findings demonstrate that positive beliefs about telemedicine are significant predictors for its frequent uptake in the future. Given that mental health providers want to use telemedicine more frequently in their practice, there is a significant need to identify methods to help them achieve this goal. In this study, providers indicated that this could be achieved by receiving assistance to coordinate health insurance reimbursements, manage a successful telemedicine practice, and facilitate a better integration of telemedicine into other health IT solutions. Research is needed to develop interventions that leverage these useful determinants of telemental health acceptability to increase providers’ weekly use of telemedicine. Telemedicine has the potential to revolutionize mental health care delivery; therefore, optimizing its routine use among its gatekeepers is a priority.

## Figures and Tables

**Table 1:** Characteristics of telemental health providers, *N* = 177.

Demographic Characteristics	*M* (*SD*) *or n* (%)
**Age**, *M (SD)*	46.4 (12.2)
**Sex**, *n* (%)	
Female	122 (68.9)
Male	55 (31.1)
**Race**, *n* (%)	
White	138 (78.0)
More than one race	13 (7.3)
Black or African American	13 (7.3)
Asian	11 (6.2)
American Indian/Alaska Native	1 (0.6)
Unknown	1 (0.6)
**Ethnicity**, *n* (%)	
Hispanic	15 (8.5)
Not Hispanic/Latino	160 (90.4)
**Highest education level**, *n* (%)	
Master’s degree	113 (63.8)
Doctor of Philosophy (PhD)	27 (15.3)
Doctor of Psychology (PsyD)	18 (10.1)
Medical Doctor/Doctor of Osteopathic Medicine (MD/DO)	12 (6.7)
Bachelor’s degree	3 (1.7)
Doctor of Nursing Practice (DNP)	2 (1.1)
Doctor of Pharmacy (PharmD)	1 (0.6)
Other	2 (1.1)
**Professional Characteristics**	***M* (*SD*) *or n* (%)**
**Years providing mental health care**, *M* (*SD*)	13.6 (10.4)
**Years providing telemental health care**, *M (SD)*	2.1 (1.9)
**Provider specialty**, *n* (%)	
Mental health counselor (LMHC, LPC)	58 (32.8)
Psychologist (PhD, PsyD)	39 (22.0)
Social worker (LCSW)	37 (20.9)
Clinical provider (Other)	21 (11.9)
Primary care provider (MD, NP, Physician Assistant or PA)	9 (5.1)
Psychiatrist (MD)	5 (2.8)
Non-clinical professionals (e.g., health coach)	3 (1.7)
Specialty physician	4 (2.3)
Therapy/rehabilitation provider	1 (0.6)
**Type of practice**, *n* (%)	
Individual practice	123 (69.5)
Part of a small clinic	32 (18.1)
Part of a network of providers	13 (7.3)
College or university	2 (1.1)
Hospital or large health organization	2 (1.1)
Primary/secondary school (Kindergarten – Grade 12)	1 (0.6)
Other	4 (2.3)

**Table 2:** Telemedicine use among mental health providers, *N* = 177.

Characteristics of Telemedicine Use	*n*	%
**Percentage of work week spent providing care to patients**		
<25%	26	14.7
25–50%	28	15.8
50–75%	47	26.6
>75%	75	42.4
Missing	1	0.6
**Percentage of time providing care to patients via telemedicine**		
<25%	132	74.6
25–50%	19	10.7
50–75%	12	6.8
>75%	14	7.9
**Patterns of telemedicine use**		
Use telemedicine only when necessary	83	46.9
Routine use a combination of in-person and telemedicine appointments	78	44.1
Use telemedicine for all appointments	16	9.0
**Billing insurance for telemedicine visits**		
Bill insurance	90	50.6

**Table 3:** Perceived benefits and barriers of telemedicine in clinical practice, *N* = 177.

Perceived Benefits and Barriers of Telemedicine	*n*	%
**Perceived benefits**		
Can see patients wherever they are	128	72.3
Can reach patients who otherwise wouldn’t receive care	119	67.2
Can have a better work life balance	67	37.9
Can be at the forefront of innovative care	57	32.2
Can see more patients and expand practice	50	28.2
Can provide care the way they want, on their terms	50	28.2
Can cut expenses	49	27.7
Other	11	6.2
**Perceived barriers**		
Technology problems	116	65.5
Security/ privacy concerns	66	37.3
Reimbursement issues	60	33.9
Legal concerns	55	31.1
Patient satisfaction	52	29.4
Competition	5	2.8
Other	9	5.1

**Table 4:** Reasons for telemedicine use, important features, and intentions for its future use, *N* = 177.

Telemedicine Features and Intentions for its Future Use	*n*	%
**Reasons for choosing a telemedicine platform**		
HIPAA compliance	157	88.7
Cheaper than other solutions	100	56.5
Features that were offered	72	40.7
Recommended by colleagues	68	38.4
Easy to use	64	36.2
Didn’t look at other options	29	16.4
Customer support available	13	7.3
Organization required it	9	5.1
**Most important features of a telemedicine platform**		
HIPAA compliant and secure	144	81.4
Free/low cost	119	67.2
Simple and easy for patients	111	62.7
Simple and easy for providers	59	33.3
No downloads needed	54	30.5
Workflow (e.g., check in, waiting room, patient queue)	32	18.1
Extensions (e.g., payment, screenshare, group call)	3	1.7
**Telemedicine features that need to be improved**		
Improve audio/video quality	85	48
Improve technical issues and bugs	66	37.3
More video-call features (recording, transcription, whiteboard)	52	29.4
Include practice management functionality (scheduling, notes)	36	20.3
Help coordinating insurance reimbursements	33	18.6
Help becoming more successful with telemedicine practice	32	18.1
More reassurance regarding legal and security concerns	29	16.4
Better integration with other health IT software I use	20	11.3
Help me connect and network with other telemedicine providers	18	10.2
Provide better customer support	10	5.6
Make it easier to use	8	4.5
**Intentions to use telemedicine in the future**		
I expect to use it more	126	71.2
I expect to use it about the same	50	28.2
I expect to use it less	1	0.6

Note. Health Insurance Portability and Accountability Act (HIPAA).

**Table 5:** Regression for benefits of telemedicine on intentions to use telemedicine in the future.

Benefits of Telemedicine	OR	95% CI	
		LL	UL
**Can see patients wherever they are**			
Not Selected	1		
Selected	3.27	0.60	17.80
**Can reach patients who otherwise wouldn’t receive care**			
Not Selected	1		
Selected	5.99 *	1.20	29.80
**Can have a better work-life balance**			
Not Selected	1		
Selected	7.13 *	1.51	33.73
**Can be at the forefront of innovative care**			
Not Selected	1		
Selected	5.83 *	1.30	26.26
**Can see more patients and expand my practice**			
Not Selected	1		
Selected	9.27 **	1.91	44.98
**Can provide care the way I want, on my terms**			
Not Selected	1		
Selected	6.46 *	1.42	29.42
**Can cut my expenses**			
Not Selected	1		
Selected	7.40 *	1.54	35.46

Note. *N* = 176; OR = Odds Ratio; LL = Lower Level and UL = Upper Level; * *p* < 0.05; ** *p* < 0.001.

**Table 6:** Regression of telemedicine features that need improvement on intentions for its future use.

Features Needing Improvement	OR	95% CI	
		LL	UL
**Improve audio/video quality**			
Not Selected	1		
Selected	0.67	0.30	1.48
**Improve technical issues and bugs**			
Not Selected	1		
Selected	0.60	0.27	1.35
**More video-call features (recording, transcription, whiteboard)**			
Not Selected	1		
Selected	1.75	0.75	4.09
**Include practice management functionality (scheduling, notes)**			
Not Selected	1		
Selected	0.81	0.27	2.37
**Help coordinating insurance reimbursements**			
Not Selected	1		
Selected	4.29 *	1.16	15.89
**Help becoming more successful with telemedicine practice**			
Not Selected	1		
Selected	13.17 *	1.57	110.82
**More reassurance regarding legal and security concerns**			
Not Selected	1		
Selected	0.72	0.25	2.07
**Better integration with other health IT software I use**			
Not Selected	1		
Selected	5.45 *	1.12	26.39
**Help me connect and network with other telemedicine providers**			
Not Selected	1		
Selected	1.75	0.18	16.69
**Provide better customer support**			
Not Selected	1		
Selected	0.96	0.19	4.80
**Make it easier to use**			
Not Selected	1		
Selected	6.21	0.68	56.41

Note. *N* = 176; OR = Odds Ratio; 95% Confidence Interval [CI] LL = Lower Level and UL = Upper Level. * *p* < 0.05.
